# 柱前衍生-高效液相色谱-荧光检测法测定白酒中的甜蜜素

**DOI:** 10.3724/SP.J.1123.2023.10016

**Published:** 2024-08-08

**Authors:** Changyu GU, Xiaobin LI, Xiao CHAI, Zhen WANG, Chenyu HOU, Runhui KE

**Affiliations:** 1.中轻检验认证有限公司, 北京 100016; 1. Sinolight Inspection & Certification Co., Ltd., Beijing 100016, China; 2.中国食品发酵工业研究院有限公司, 北京 100015; 2. China National Research Institute of Food & Fermentation Industries Co., Ltd., Beijing 100015, China

**Keywords:** 高效液相色谱, 荧光检测, 柱前衍生, 甜蜜素, 白酒, high performance liquid chromatography (HPLC), fluorescence detection, pre-column derivatization, sodium cyclamate, Baijiu

## Abstract

白酒中甜蜜素(化学名为环己基氨基磺酸钠)是我国食品安全监督抽检计划中的重点关注项目,生产企业亟需简单、经济、灵敏度高而且可靠的检测方法。本研究建立了柱前衍生-高效液相色谱-荧光检测法测定白酒中甜蜜素的方法。在酸性条件下,通过脱磺酸基反应将样品中的环己基氨基磺酸钠转化为氨基化合物,然后加入400 g/L的氢氧化钠溶液中和样品处理液,再加入邻苯二甲醛衍生试剂与样品溶液中的氨基化合物进行反应,产生具有荧光信号的吲哚取代衍生物。以反相C18色谱柱(250 mm×4.6 mm, 5 μm)为分析柱,乙腈和磷酸盐缓冲液为流动相进行等度洗脱,荧光检测器检测,外标法定量。结果表明,甜蜜素在0.1~2.0 mg/L范围内呈良好的线性关系,其相关系数(*r*^2^)大于0.999。基于白酒样品,在0.1~1.0 mg/kg含量范围内进行3个浓度水平的加标回收试验(*n*=6),平均回收率为90.7%~100.9%,相对标准偏差为3.5%~5.6%,方法的检出限为0.03 mg/kg,定量限为0.10 mg/kg。应用本方法对市售9种白酒样品进行检测,结果与GB 5009.97-2016(第三法)液相色谱-串联质谱法所得结果一致。本方法具有成本低、灵敏度高、专属性强、定量结果准确的特点,适用于大批量白酒中甜蜜素的定量检测,可为生产企业或相关机构进行白酒中甜蜜素的日常监测提供有力的技术支持。

## Abstract (en)

Sodium cyclamate in Baijiu is a key item in the China National Food Safety Supervision and Inspection Plan. A simple, economical, sensitive, and reliable method is urgently needed for routine analysis and internal quality control. A method based on high performance liquid chromatography with fluorescence detection (HPLC-FLD) was developed for the determination of sodium cyclamate in Baijiu by *o*-phthalaldehyde derivatization. First, the sodium cyclamate in the sample solution was converted into amino compounds using the desulfurization reaction under acidic conditions. Next, 400 g/L sodium hydroxide solution was added to the sample solution for neutralization. The amino compounds in the sample solution were then derivatized with *o*-phthalaldehyde to produce indole-substituted derivatives that are capable of producing fluorescence signals. Separation was carried out on a C18 column (250 mm×4.6 mm, 5 μm) in isocratic elution mode using a mobile phase consisting of acetonitrile and phosphate buffer. Finally, the eluate was monitored using a fluorescence detector, and an external standard method was used for quantification. A good linear relationship was obtained in the range of 0.1-2.0 mg/L, with correlation coefficients greater than 0.999. The average recoveries of sodium cyclamate spiked at levels of 0.1-1.0 mg/kg in Baijiu samples ranged from 90.7% to 100.9%, with relative standard deviations (RSDs) of 3.5%-5.6% (*n*=6). The limits of detection and quantification were 0.03 and 0.10 mg/kg, respectively. Nine Baijiu samples collected from the market were tested, and the results demonstrated that the contents of sodium cyclamate detected by the developed method were consistent with those obtained using the liquid chromatography-tandem mass spectrometry (LC-MS/MS) method described in GB 5009.97-2016 (the third method). The proposed method is economical, sensitive, specific, and accurate; thus, it provides a basic approach for the determination of sodium cyclamate in Baijiu samples and has great potential for routine analysis in foodstuffs.

甜蜜素,化学名为环己基氨基磺酸钠,是一种人工合成甜味剂,甜度为蔗糖的30~40倍,是食品工业中常用的添加剂^[1]^。在长期的研究和临床实践中发现,过量摄入甜蜜素会对人体的代谢系统、神经系统等造成影响,且存在一定的致癌风险^[2,3]^。目前世界上对甜蜜素的安全性仍存在争议,美国、日本等40多个国家禁止使用甜蜜素作为食品甜味剂^[4]^,但中国、欧盟等80多个国家和地区则允许使用^[1]^。白酒是我国特有的蒸馏酒,历史悠久、工艺独特,是中国饮食文化的象征之一^[5]^。优质白酒本身含有一定量的多元醇等甜味物质,所以其味道醇厚,且有绵甜之感^[6]^,这有赖于一定的酿造时间、精良的酿造技术以及优质的原料。但是,一些酒企为了改善白酒风味,快速、低成本实现优质酒独有的“回甘”特点,向白酒中添加甜蜜素等甜味剂^[7]^。GB/T 15109-2021《白酒工业术语》规定了白酒“不直接或间接添加非自身发酵产生的呈色呈香呈味物质”^[8]^。

目前,甜蜜素检测常用的方法主要有气相色谱法^[9]^、液相色谱-紫外检测法^[7]^、液相色谱-二极管阵列法^[10]^、液相色谱-蒸发光散射法^[11]^、液相色谱-串联质谱法^[12,13]^、离子色谱法^[14]^等。其中气相色谱法已被证明不适用于白酒基质的检测,易造成假阳性结果^[15]^。液相色谱-紫外检测法和蒸发光散射法的灵敏度较低,无法满足酒类抽检的要求。离子色谱法则存在灵敏度较低、分析成本较高的缺点。目前,我国食品安全监管方案中常将液相色谱-串联质谱法作为酒中甜蜜素检测的首选方法^[16]^。但是,该仪器价格昂贵,对操作人员要求高,在大多数的中小型酒企中普及难度大^[17]^。因此,开发适用于白酒中甜蜜素检测的低成本、高灵敏度、强专属性的方法十分必要。

衍生化技术是色谱分析中常用的辅助手段,可以解决目标物保留性差、不稳定、没有检测响应等问题,在不增加仪器硬件成本的情况下,衍生化不失为一种较有效的分析手段^[18]^。荧光衍生化试剂能与一些官能团产生强烈荧光,在较温和条件下与被测物质快速定量反应,尤其适合痕量分析。荧光检测器是目前液相色谱法中灵敏度最高的检测器之一,特异性强,与质谱检测器检出浓度相当,是进行白酒中甜蜜素痕量分析的一种较理想的检测方法,但目前未见相关应用。甜蜜素本身不产生荧光,使用荧光检测法需要采用合适的衍生试剂将甜蜜素衍生,同时要避免白酒中环己醇及环己基类似物产生的干扰^[19]^。

基于上述问题,本研究通过优化前处理和仪器检测条件,建立了一种柱前衍生-高效液相色谱-荧光检测器法测定白酒中甜蜜素的方法,方法的检出限、回收率、精密度及抗干扰能力均符合相关检测要求,用于实际样品的检测可满足低成本、高灵敏度和强专属性的检测需求,大大降低了应用门槛。

## 1 实验部分

### 1.1 仪器、试剂与材料

LC-20A液相色谱仪(日本岛津公司); HWS-26电热恒温水浴锅(上海一恒科学仪器有限公司);超声波清洗器(昆山超声仪器有限公司); Milli-Q Reference超纯水器(美国Millipore公司); 0.22 μm尼龙滤膜(上海安谱科学仪器有限公司)。

环己基氨基磺酸钠标准物质(纯度99.9%, CAS号:139-05-9,批号:2254009)购自上海安谱璀世标准技术服务有限公司。甲醇、乙腈(色谱纯,美国J. T. Baker公司);乙酸、乙酸铵(HPLC级,北京迪马科技有限公司);磷酸氢二钠、过氧化氢溶液和磷酸二氢钠均为分析纯,购自西陇化工股份有限公司;磷酸、氢氧化钠和浓盐酸均为分析纯,购自北京化工厂;邻苯二甲醛(OPA)和3-巯基丙酸购自阿法埃莎(中国)化学有限公司。

### 1.2 标准溶液及试剂的配制

准确称取0.100 g环己基氨基磺酸钠标准品,置于100 mL容量瓶中,用超纯水配制成质量浓度为1.0 g/L的标准储备液,于4 ℃冰箱冷藏保存。准确吸取10 mL标准储备溶液于100 mL容量瓶中,用超纯水配制成质量浓度为100 mg/L的标准工作液,于4 ℃冰箱中冷藏保存。

OPA衍生试剂:200 mg邻苯二甲醛用5 mL乙醇溶解,加入1 mL 3-巯基丙酸,用pH=10.0硼酸盐缓冲液定容至100 mL(反应16 h后再使用,有效期不超过10天,于0~4 ℃暗处储存)。

### 1.3 样品前处理方法

将2.00 g白酒样品置于蒸发皿中于95 ℃水浴锅上蒸发至干,然后用2 mL 90%甲醇水溶液溶解。转移至10 mL具塞比色管中,加入1 mL过氧化氢溶液,再加入0.3 mL浓盐酸,涡旋混匀1 min,在100 ℃恒温水浴中放置60 min。取出比色管,冷却至室温,加入0.4 mL 400 g/L的NaOH溶液,混匀,然后加入OPA衍生溶液至10 mL刻度线,混匀,反应5 min后过0.22 μm滤膜,供液相色谱-荧光检测器测定。

### 1.4 液相色谱-荧光检测条件

色谱柱:C18柱(250 mm×4.6 mm, 5 μm,美国Waters公司);流动相:乙腈-磷酸盐缓冲液(64∶36, v/v);等度洗脱;流速:1.0 mL/min;柱温:40 ℃;进样量:10 μL;激发波长:350 nm;发射波长:440 nm。

## 2 结果与讨论

### 2.1 色谱条件的选择

流动相是影响高效液相色谱分离性能的重要因素。本研究对比了乙腈和甲醇作为有机相时的出峰情况。实验表明,采用乙腈和甲醇时均能达到良好的出峰效果,但乙腈的洗脱能力更强,柱压更低,且乙腈吸光度值低,基线噪声更小,为了兼顾分离和检测时间,本实验采用乙腈为有机相。甜蜜素水溶液pH为5.5~7.5,呈弱酸性,因此考虑采用弱酸性水相流动相来抑制目标组分的解离,增加组分在色谱固定相上的保留,进而改善色谱峰形,实现更好的分离。磷酸盐缓冲液具有短波长区低吸收以及宽的pH缓冲范围的特点,常作为甜蜜素测定时的水相流动相。因此,实验考察了流动相中乙腈和磷酸盐缓冲液的不同配比对甜蜜素色谱分离的影响。结果表明:乙腈和磷酸盐缓冲液的比例为64∶36(v/v)时,目标物质与干扰峰完全分离,保留时间9.517 min,峰形较好。采用等度洗脱节省了测样时柱平衡的时间,且柱压更为稳定,克服了梯度洗脱时造成的基线漂移。另外,实验进一步考察了不同流速对出峰情况的影响,结果表明,流速对峰的分离效果影响不大,但流速慢则出峰时间长,最终选定甜蜜素的测定流速为1.0 mL/min。甜蜜素标准溶液(0.5 mg/L)的色谱图见图1。

**图1:**
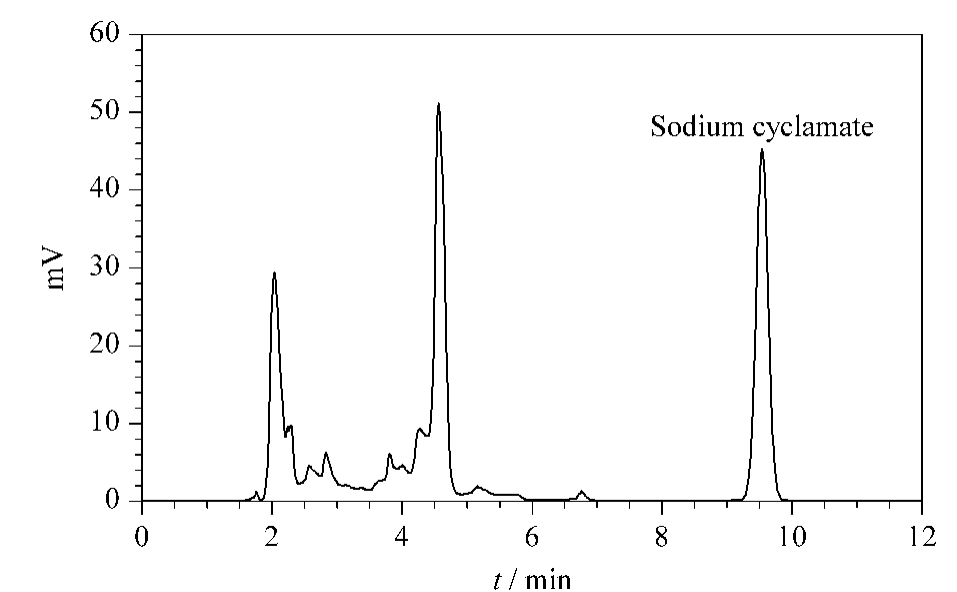
甜蜜素标准溶液(0.5 mg/L)的色谱图

### 2.2 样品前处理条件的优化

#### 2.2.1 荧光衍生试剂的选择

荧光衍生试剂可以改善目标物的检测性能。在液相色谱分析中,荧光检测器的灵敏度比紫外检测器高10~1000倍,但是自然界中大多数化合物不产生荧光,通过衍生化反应在目标物上加成能发出荧光的生色基团,就可以达到荧光检测的目的,由于荧光衍生物的激发波长、发射波长与衍生化试剂的不同,即使衍生化试剂过量或者有副反应,也不干扰荧光衍生物的检测。

衍生化反应的关键是衍生试剂的选择。目前,液相色谱分析常用的荧光衍生化试剂主要有邻苯二甲醛、荧光胺、丹酰氯、芴代甲氧基酰氯等^[20]^。在含氨基的化合物检测中,荧光衍生化试剂使用最多、衍生条件最成熟的是邻苯二甲醛^[21,22]^,具有流程简单、副产物少、灵敏度高的衍生特点^[23]^,其荧光反应机理是利用没有荧光信号的邻苯二甲醛在碱性条件下与氨或铵离子反应产生具有荧光信号的吲哚取代衍生物,其荧光强度与氨或铵离子的含量成正比^[24]^。因此本实验选择邻苯二甲醛作为荧光衍生剂。

#### 2.2.2 脱磺酸基反应的优化

甜蜜素分子结构中含有环己基和氨基磺酸基团,可在酸性条件下经脱磺酸基反应水解形成环己胺^[16]^,具备与邻苯二甲醛进行衍生反应生成具有强荧光的吲哚取代衍生物的结构基础。甜蜜素的脱磺酸基反应通常在酸性和加热条件下进行,而人体胃酸的主要成分为盐酸,因此本实验选择加入盐酸,并考察了不同加入量(0.1、0.3、0.5 mL)以及不同反应时间(30、45、60、90、120 min)对环己基氨基磺酸钠转化为氨基化合物的影响。结果表明:加入0.3 mL浓盐酸时反应效率最佳,且反应效率随着提取时间的延长而增加,60 min时最高,60 min以后反应效率趋于稳定(图2)。

**图2:**
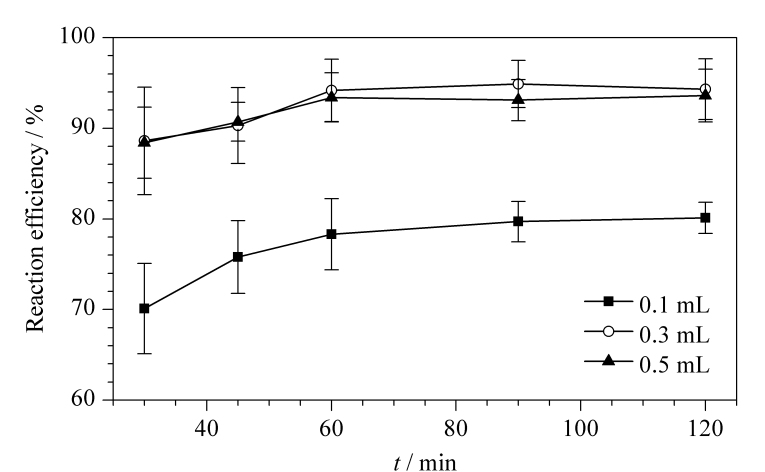
盐酸用量及反应时间对甜蜜素反应效率的影响(*n*=3)

#### 2.2.3 中和反应的优化

邻苯二甲醛需要在碱性条件下与氨/铵离子反应产生具有荧光信号的吲哚取代衍生物,在实验过程中需要添加一定量的碱性溶液以去除脱磺酸基反应中添加的酸,同时保持反应在碱性条件下进行,因此本实验通过比对不同体积(0.1、0.2、0.3、0.4、0.5、0.6 mL)400 g/L氢氧化钠的加入量以确定最佳的中和反应条件。结果表明:加入0.4 mL 400 g/L的NaOH溶液,反应效率最佳(图3)。

**图3:**
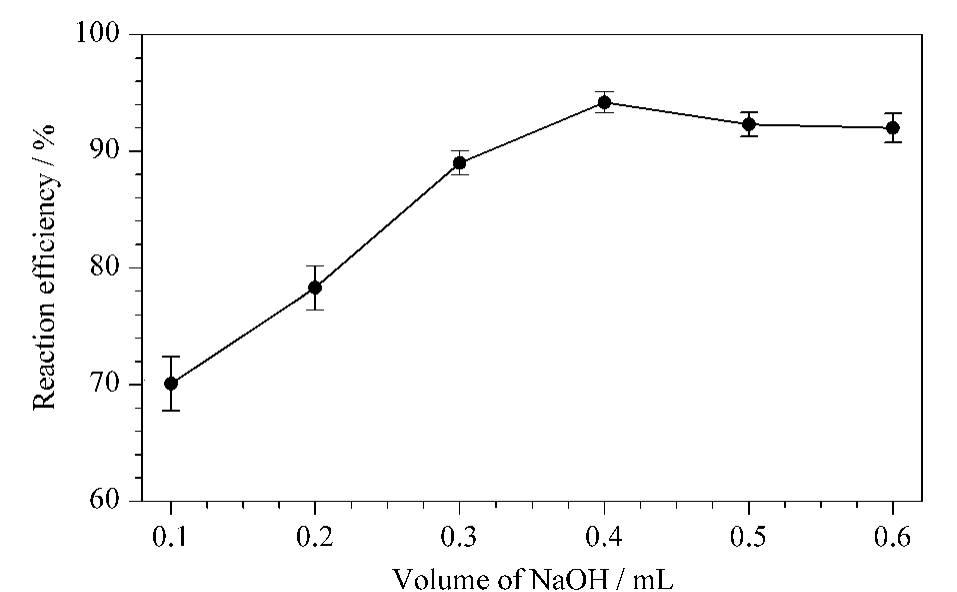
NaOH用量对甜蜜素反应效率的影响(*n*=3)

#### 2.2.4 衍生试剂用量的优化

衍生试剂用量可能会对甜蜜素的结果产生影响,因此本实验对比了不同体积OPA试剂对衍生反应效果的影响。测定结果表明:当OPA体积为6~10 mL时,白酒中甜蜜素的反应效率均在90.0%以上(图4)。当OPA体积6 mL时,反应效率最佳,为93.8%。本实验从方便计算的角度考虑,利用加入的OPA溶液将反应体系体积定容至10 mL,此时OPA试剂加入体积约为6.3 mL。

**图4:**
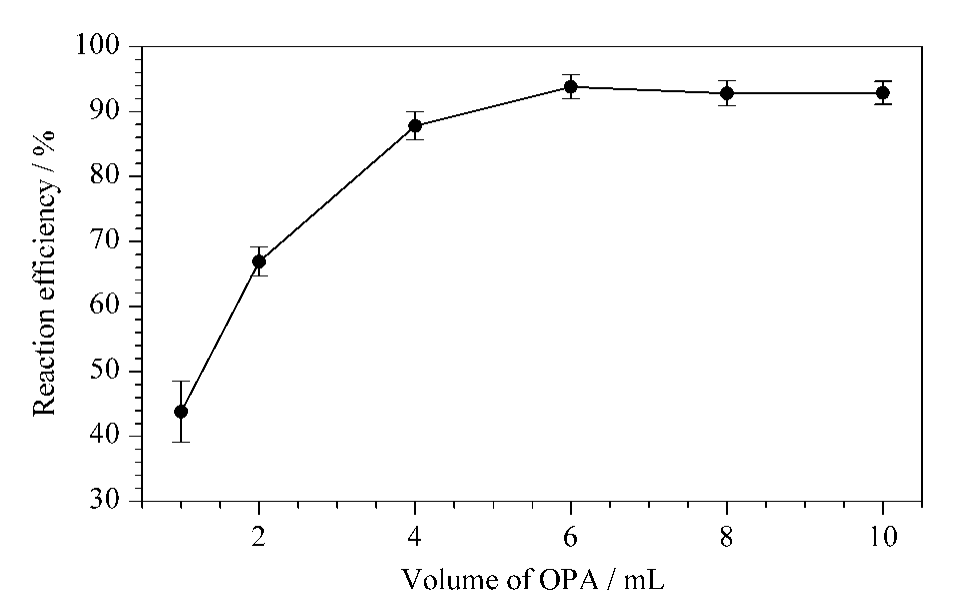
OPA用量对甜蜜素反应效率的影响(*n*=3)

### 2.3 衍生试剂与衍生物的稳定性

OPA容易被空气中的氧气所氧化,从而发生降解,因此应该在使用期限内使用衍生试剂。选取不同日期配制的OPA衍生试剂(0~4 ℃避光保存)进行衍生化反应,发现当采用储存时间10天以内的OPA试剂进行实验时,甜蜜素测定结果相差不大(图5)。因此,确定配制好的OPA衍生试剂使用有效期不超过10天。

**图5:**
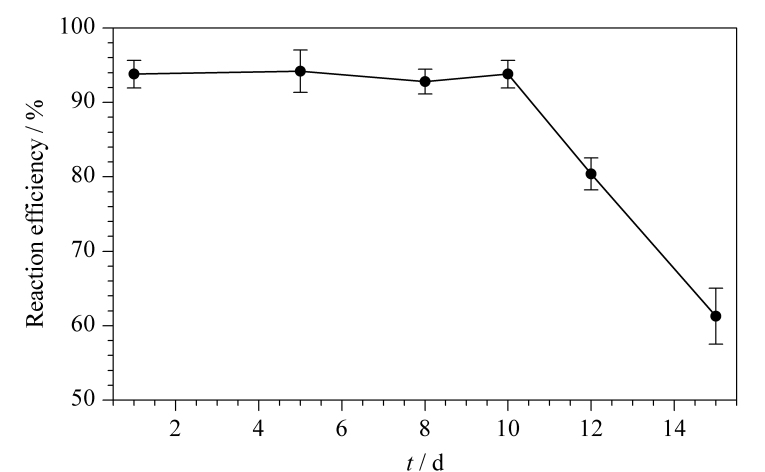
衍生剂储存时间对甜蜜素反应效率的影响(*n*=3)

### 2.4 溶解溶液对甜蜜素衍生物稳定性的影响

OPA衍生具有反应速度快的优点,但也有衍生物稳定性差的缺点^[25]^。实验结果表明,白酒中甜蜜素直接衍生,其衍生物的半衰期只有0.5 h,衍生后需迅速检测,无法满足批量样品的检测需求。汪建飞等^[25]^发现,*γ*-氨基丁酸与OPA衍生物的降解速度与衍生体系中甲醇浓度成反比。因此,本实验考察了不同溶解溶液对甜蜜素衍生物稳定性的影响。

分别取适量的甜蜜素标准工作液用纯水和30%、50%、90%甲醇水溶液定容至2 mL,按1.3节方法衍生化后制备成0.1 mg/L的甜蜜素标准溶液,分别取10 μL进样检测,连续进样10 h,计算甜蜜素衍生物的半衰期。

按一级动力学模型公式(1)计算甜蜜素衍生物的降解速度。


(1) $$\mathrm{lg}C=-\frac{K}{2.303}+\mathrm{lg}C_{0}$$

其中*C*为甜蜜素衍生物各时间点的含量(mg/mL); *K*为降解速率常数;*C*_0_为甜蜜素衍生物的含量(mg/mL)。

结果表明,随着溶解溶液中甲醇体积分数的增加,甜蜜素衍生物的降解速率常数*K*值不断变小(图6)。不同甲醇体积分数(0、30%、50%、90%)下甜蜜素衍生物的半衰期分别为6.7、8.7、20.5、76.7 h,说明反应体系中甲醇能够促进邻苯二甲醛、3-巯基丙酸与甜蜜素的结合^[25]^,并且增强甜蜜素衍生物的稳定性。

**图6:**
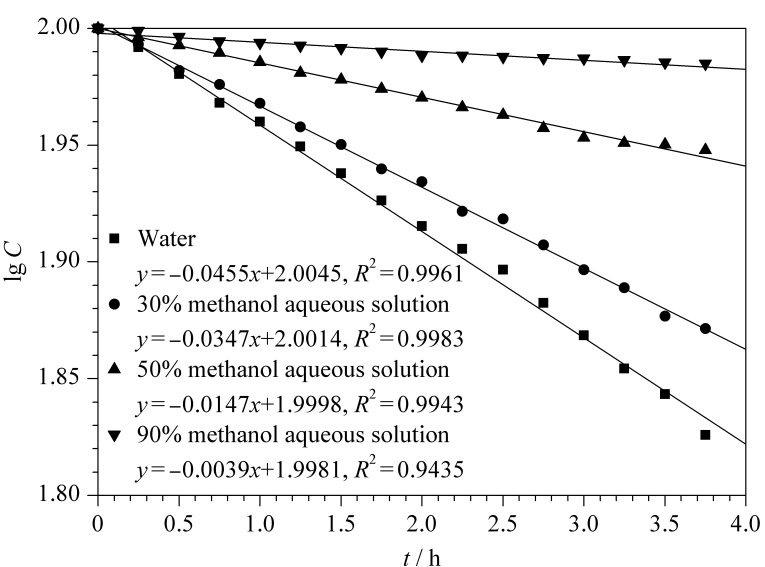
不同溶解溶液中甜蜜素衍生物(0.1 μg/mL)的lg *C*趋势图

### 2.5 系统适应性评价

由于白酒中含有其他环己醇及环己基类似物,与甜蜜素分子结构中的环己基和氨基磺酸基团接近,这些物质可能会干扰衍生反应,从而对实验结果产生影响,因此需要进行方法的专属性考察。本实验选择未检出甜蜜素的白酒样品进行加标,考察测定结果是否受到干扰。如图7所示,空白样品在甜蜜素的出峰位置无干扰峰,故白酒中环己醇及环己基类似物不会对甜蜜素的检测结果产生影响。

**图7:**
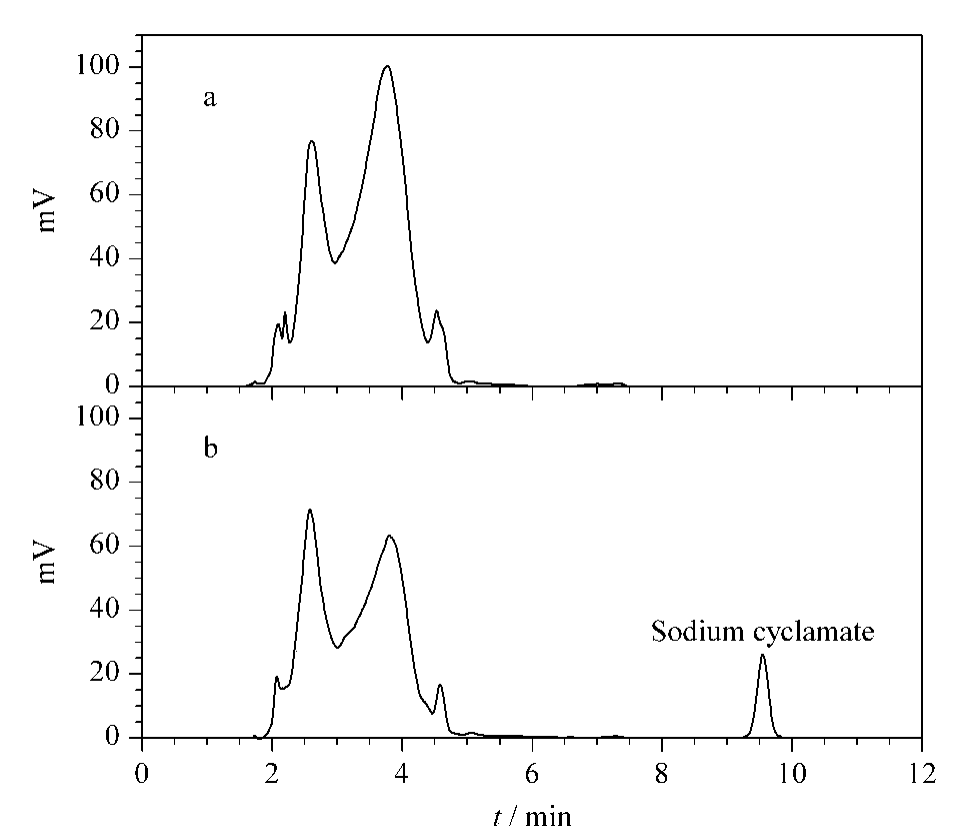
白酒样品加标(a)前、(b)后的色谱图

### 2.6 线性范围、检出限和定量限

分别取一定量的甜蜜素标准工作溶液,用90%甲醇水溶液定容至2.0 mL,按照样品处理方法进行衍生化反应,制备成0.1、0.2、0.5、1.0、2.0 mg/L的标准工作溶液,在优化色谱条件下检测,以峰面积(*Y*)和对应的质量浓度(*X*)绘制标准曲线,得到的线性方程为*Y*=1178752.2*X*-45649.2(相关系数*r*^2^=0.9998)。结果显示,甜蜜素在0.1~2.0 mg/L范围内呈良好的线性关系。方法的检出限(LOD)和定量限(LOQ)采用向空白样品中逐级降低加标浓度的方法来确定。以3倍信噪比(*S/N*=3)对应的目标物含量作为检出限,以*S/N*=10对应的目标物含量作为定量限,分别为0.03 mg/kg和0.10 mg/kg,与GB 5009.97-2016(第三法)一致。

### 2.7 回收率和精密度

分别在空白白酒样品中添加低、中、高3个水平的甜蜜素标准溶液,每个水平平行测定6次,计算回收率和精密度。结果表明,甜蜜素平均回收率为90.7%~100.9%,相对标准偏差为3.5%~5.6%(见表1),可用于白酒样品中甜蜜素含量的测定。

**表1:** 白酒中甜蜜素的加标回收率和相对标准偏差(*n*=6)

Spiked/(mg/kg)	Recovery/%	RSD/%
0.1	90.7	5.6
0.2	94.2	3.5
1.0	100.9	4.2

### 2.8 实际样品的测定

对市售9种白酒样品进行含量测定。图8为1号阳性样品的色谱图。同时,采用GB 5009.97-2016第三法液相色谱-串联质谱法进行测定。每个样品重复测定3次,检测结果见表2。可以看出本方法与GB 5009.97-2016(第三法)检测结果一致。

**图8:**
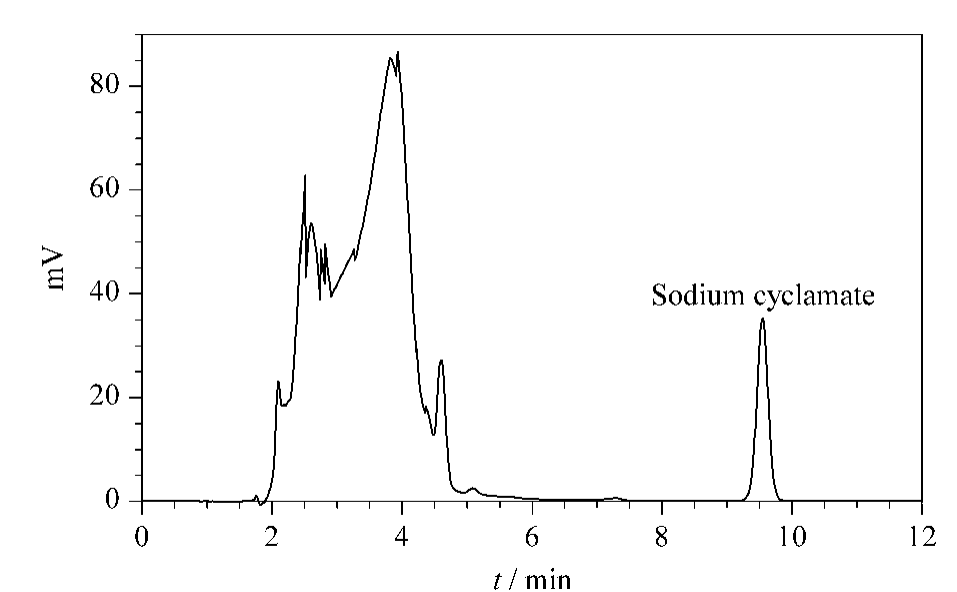
某阳性白酒样品中甜蜜素的色谱图

**表2:** 市售白酒样品中甜蜜素的检测结果(*n*=3)

No.	HPLC-FLD			GB 5009.97-2016	
	Content/(mg/kg)	RSD/%		Content/(mg/kg)	RSD/%
1	1.23	2.1		1.20	1.9
2	<LOQ	/		<LOQ	/
3	<LOQ	/		<LOQ	/
4	<LOQ	/		<LOQ	/
5	<LOQ	/		<LOQ	/
6	0.120	1.7		0.110	1.8
7	<LOQ	/		<LOQ	/
8	<LOQ	/		<LOQ	/
9	<LOQ	/		<LOQ	/

## 3 结论

本研究建立了柱前衍生-液相色谱-荧光检测测定白酒中痕量甜蜜素的定量分析方法,通过荧光衍生化试剂在目标物上连接能发出荧光的生色基团,达到荧光检测的目的,提高了方法的灵敏度。该方法具有操作简单、基质干扰小、成本低、灵敏度高等特点,可以满足白酒生产企业或相关机构在缺乏液相色谱-串联质谱仪的情况下实现批量白酒样品中甜蜜素快速分析的要求,具有较好的实用价值。
